# Pediatric Thermal Burns and Treatment: A Review of Progress and Future Prospects

**DOI:** 10.3390/medicines4040091

**Published:** 2017-12-11

**Authors:** Elton Mathias, Madhu Srinivas Murthy

**Affiliations:** 1 Department of Clinical Research, Mallinckrodt Pharmaceuticals, Bedminster, NJ 07921, USA; 2 Centre for Applied Genetics, Bangalore University, Bangalore 560056, India; madhu.srinivas06@gmail.com

**Keywords:** burns, treatment, pediatric, autograft, biological skin substitute, thermal

## Abstract

Burn injuries are a devastating critical care problem. In children, burns continue to be a major epidemiologic problem around the globe resulting in significant morbidity and death. Apparently, treating these burn injuries in children and adults remains similar, but there are significant physiological and psychological differences. The dermal layer of the skin is generally thinner in neonates, infants, and children than in adults. Enhanced evaporative loss and need for isotonic fluids increases the risk of hypothermia in the pediatric population. The pain management of the children with major burns challenges the skills of the personnel of every unit. Managing these wounds requires intensive therapeutic treatment for multi-organ dysfunction, and surgical treatment to prevent sepsis and other complications that further delay wound closure. Alternatives to the practice of donor site harvest and autografting for the treatment of severe burns and other complex skin defects are urgently needed for both adult and pediatric populations. This review article focuses on thermal burn pathophysiology and pain management and provides an overview of currently approved products used for the treatment of pediatric burn wounds. A new promising approach has been presented as a first-line therapy in the treatment of burns to reduce surgical autografting in pediatric patients.

## 1. Introduction

Approximately one-third of burn injuries in the United States (U.S.) occur in the pediatric population. Pediatric burns resulting in the hospitalization of subjects under 5 years of age are most frequently caused by scalding, whereas fire and flame injuries are a more common etiology amongst older pediatric subjects [1,2]. Each day, about 300 pediatric burn-related injuries are treated in emergency rooms [3]. Of the approximately 105,000 burn injuries reported in the U.S. in 2015 to individuals under the age of 18, 9% were non-fatal injuries involving hospitalization or transfer. Depending upon the extent of the burn, hospitalization can be protracted. Amongst infants, children, and adolescents aged 1–17 in the U.S. in 2015, fires and burns were the sixth leading cause of non-fatal unintentional injuries leading to hospitalizations and transfers [4]. Fires and burns are currently the fifth leading cause of deaths in the United States that occur in the home, the third leading cause of unintentional injury-related fatalities among children and adolescents aged 5–14, and the fourth most prevalent cause for infants and children aged 1–4 [5]. National preventive measures and education efforts have successfully lowered the number of burns in the United States [6]. The Centers for Disease Control and Prevention (CDC) has implemented a National Action Plan in order to raise awareness and reduce the numbers of pediatric burn injuries by targeting six areas including data and surveillance, research, communication, education and training, health systems and healthcare, and policy [7]. Efforts include teaching families about lowering water heater temperatures, testing bath temperatures, and raising water heaters off the ground to prevent house fires.

Managing these wounds requires intensive therapeutic treatment for multi-organ dysfunction, and surgical treatment to prevent sepsis and other complications. In children, burns continue to be a major epidemiologic problem around the globe resulting in significant morbidity and death [8]. Recent advancement shows improved pain management resulting in diminished mortality. This review focuses on current advancements in wound healing and pain care management in pediatric patients.

## 2. Background

### 2.1. Skin Function

As the interface with the external environment, the skin serves as an effective barrier to chemicals, toxins, and irritants. Intact skin functions to prevent local infection of the dermis or other underlying tissue by microorganisms (e.g., bacteria, fungi, and other pathogens). Skin also regulates temperature and fluid homeostasis, serving as a barrier to the loss of water vapor and heat from the body. For individuals with significant skin loss, disruption of cutaneous barrier function not only results in a continuous risk of life-threatening infection but also mandates continuous fluid resuscitation and protection from environmental toxins [9].

**Figure 1:**
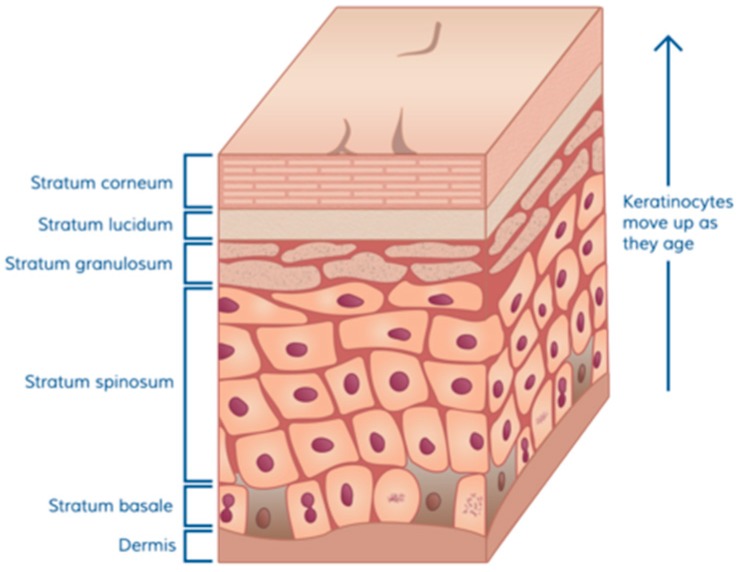
Cuboidal cells above the dermal matrix representing the basal cells.

Normal skin is composed of three basic parts: a dermal matrix composed of collagen and other extracellular matrix glycoproteins that provides resiliency and elasticity and is constantly restored by dermal fibroblasts, an overlying epidermal layer with the outermost layer containing a thick oily layer of desquamating cells that prevent water loss and exposure to foreign invaders, and skin appendages including hair follicles, sweat glands, Merkel cells, and Langerhans crypts which contain immunological cells. The skin of full-term infants contains the same layers as are present in adult skin, however, the infant dermis is thin and steadily increases in thickness from infancy to puberty [10]. In native skin, the epidermis is attached to the dermis via a thick basement membrane which is produced by the basal keratinocytes. The cuboidal cells shown above the dermal matrix in Figure 1 [11] represent the basal cells that are the continuous source, through cell division, of the upper layers of the epidermis. As basal keratinocytes divide, some undergo differentiation during which specific proteins and lipids needed to generate an epidermal permeability barrier are produced [12].

The barrier function of the skin is dependent on the differentiation of keratinocytes to generate mature squames (flattened cells at the top of Figure 1). This process includes the assembly of highly cross-linked proteins into a cornified envelope beneath the plasma membrane, secretion of lipids into the intercellular space, and finally keratinocyte enucleation. Cells gradually die and the squames are then sloughed off by friction, cleaning, and other minor trauma. Bacteria that attempt to invade through the skin are thus captured in and among dying cells that are subsequently shed. In addition, as keratinocytes differentiate they produce host defense peptides which are a critical component of innate defenses against wound infection. These antimicrobial peptides act locally within the epidermal and stromal tissues of skin and protect against a broad range of microorganisms, fungi, and viruses [13]. During injury, keratinocytes are also a rich source of chemotactic and growth factors that are crucial to the orchestration of the immune response and wound healing [14].

Loss or disruption of skin function often results in significant mortality and morbidity, putting the individual at significant risk of extended hospitalization and death. Disruption of homeostatic and barrier functions leaves the underlying tissues highly susceptible to infection. Such local infections, when left unchecked, can quickly result in systemic infection [9].

### 2.2. Overview of Pathophysiology of Burn Wounds in Pediatric Patients

Burn pathophysiology is similar in adult and pediatric populations, however, differences in their size and metabolic state requires extra consideration in the treatment of pediatric burns. Of immediate concern in the treatment of burns is inhalation injury, which is among the most lethal aspects of burns. Subjects with burns must be monitored for carbon monoxide poisoning and acute respiratory distress syndrome (ARDS) [8]. The risk for airway compromise and likelihood for intubation is increased in pediatric subjects due to a smaller airway opening and greater risk for closure from edema [6]. In response to a burn injury, an inflammatory response occurs which is characterized by the release of catecholamines, vasoactive mediators, and inflammatory markers which can trigger the onset of systemic inflammatory release syndrome (SIRS), regardless of the age of the subject. Resulting capillary leaks induce protein loss and interstitial edema [8]. The combination of tissue injury, inflammatory response, and hypovolemia can cause shock-associated hypotension and myocardial depression [6,8]. Tachycardia is frequently observed in those with burns, regardless of age [8]. While an inflammatory response is a characteristic in both pediatric and adult populations, pediatric subjects may mount a greater reaction and are generally more vulnerable to the systemic effects. They are also more susceptible to the post-burn hypermetabolic state induced by the release of inflammatory factors [6]. In this state, catabolism increases and anabolic hormone levels decrease, causing loss of muscle and bone mineral density and content and potentially interfering with wound healing [8]. Care of subjects with burns must involve nutritional support to sustain lean body mass and to promote wound healing. The hypermetabolic state is sustained long after wound closure is achieved, with protein breakdown continuing six to nine months after the initial trauma. Despite nutritional supplementation, bone growth in pediatric subjects is delayed for two years after burn injury [6]. Additionally, a continuing need for skin growth and elasticity to accommodate growth complicates wound and scar management in pediatric burns.

Most major burns are complex and may consist of superficial, deep partial-thickness, and full-thickness injury admixed. The dermal layer of the skin is generally thinner in neonates, infants, and children than adults, steadily increasing in thickness from infancy to puberty [10,15,16]. From a given heat exposure, this contributes to a greater depth of burn injury in most children compared to non-geriatric adults [17]. For example, adult mid-dermal burns are caused by temperatures of ~82 °C (180 °F) whereas mid-dermal burns in children are likely to be caused by temperatures of ~76 °C (169 °F). Skin thicknesses vary by age, body location, ethnicity, and by individual. The thickness of skin in children is approximately 70% the thickness of adult skin [18]. Thin skin coupled with reduced subcutaneous fat stores renders the initial assessment of the depth of injury more difficult in pediatric burns [19].

Loss of skin due to burns results in concomitant loss of its barrier function. Skin loss due to burn injury impairs thermoregulation and reduces the body’s ability to retain heat and water [8]. Subjects with burns require heat conservation to prevent hypothermia as well as fluid resuscitation to compensate for fluid loss and capillary leakage. The pediatric surface area to mass ratios can be nearly three times that of adults, leading to proportionally greater evaporative fluid loss in pediatric subjects [19]. Neonates, infants, and children also have higher blood volumes relative to their mass, averaging ~80 mL/kg body weight compared to the adult average of 70 mL/kg [6]. As such, fluid resuscitation in pediatric burn cases necessitates larger volumes per unit body weight, and dextrose is often co-administered to those under 20 kg (44 lb) to avoid hypoglycemia [8,20]. Enhanced evaporative loss and need for isotonic fluids increases the risk of hypothermia in this population. Burn-associated fluid loss, protein loss, a decrease in blood volume, and SIRS can result in renal and hepatic system dysfunction and are significant concerns in both pediatric and adult populations.

## 3. Current Management of Complex Skin Defects—Autograft

Severe burns are best viewed as a continuum which may exist as a mosaic within the same wound area, requiring effective management of both full-thickness regions and those with intact dermal elements. Although some complex skin defects with intact dermal elements may heal without autografting, the time required to heal these wounds is greater than 3 weeks. During this time, these open wounds are at risk for infection and other complications that further delay wound closure. Moreover, when allowed to heal on their own, these wounds exhibit significant scarring, contracture, and loss of function. Due to the significant morbidity in terms of time associated with healing, potential infection, and scarring, coupled with poor outcomes, complex skin defects with intact dermal elements and full-thickness burns are treated as a single clinical entity [21,22,23].

After stabilization from sequelae of traumatic injury, the medical management of choice for deep partial-thickness and full-thickness burn wounds in subjects of any age is excision of non-viable tissue from the wound followed by placement of an autograft as soon as possible. Autografting involves the surgical harvest of healthy skin from an uninjured site (the donor site) and its placement on the primary wound site. Skin grafts are typically split-thickness (e.g., comprised of the epidermis and the top portion of the dermis). Although wound coverage by split-thickness autograft is the standard of care for sufficiently large or deep burns, its harvest requires a surgical procedure and results in the creation of a secondary, iatrogenic wound that is painful and susceptible to fluid loss, infection, and permanent scarring.

Pediatric subjects represent a highly-vulnerable population for which harvest of autograft is undesirable. The identification of an appropriate donor site is a critical consideration in the decision to autograft [24]. The skin of neonates, infants, and children possesses a relatively thin dermal layer, minimizing the depth of tissue that can be harvested with retention of dermis across the donor site. The selection of donor sites typically involves the identification of large, relatively planar areas of healthy skin that can be hidden by clothing to reduce the cosmetic impact of potential scarring resulting from surgical harvest of autograft. In pediatric subjects, this can be complicated by limitations in the area of available healthy skin in less contoured regions due to their relatively small total body surface area, which may be further restricted in those with extensive skin defects. In extensively burned pediatric subjects, the scalp is often used as a donor site for split-thickness skin grafts because of its relatively large surface area and ability to heal rapidly. However, harvesting autograft tissue from the scalp can result in excessive blood loss, hypertrophic scarring, scalp alopecia, and chronic folliculitis. In addition, the cosmetic outcome at both treatment and donor sites can be complicated by the subject’s growth rate. For example, the surface area of the thorax increases 14-fold from infancy to puberty. Despite these challenges, cosmesis of both treatment and donor sites can be important contributors to social development and lifelong emotional health [25].

Regardless of age, autograft donor sites become painful wounds with concomitant water vapor loss, susceptibility to infection, and formation of a permanent scar. In the youngest subjects, the risk of infection is increased if the donor site is covered or partially covered by a diaper. In addition, donor site wound care in the pediatric population is especially critical due to the need for pain control during dressing changes. Donor site wounds are typically more painful than the primary wound [26]. In the pediatric population specifically, the intense pain associated with donor site wounds must be carefully managed in order to avoid lasting psychological effects. In an effort to minimize donor site size, harvested autografts are typically meshed and expanded to cover an area larger than the donor site. However, this expansion creates a mesh pattern in the autografted area resulting in an inferior cosmetic outcome. Limited healthy skin can also necessitate sequential reharvesting of available donor sites, delaying definitive closure. This delay increases the risk of infection and scarring at the wound site and often necessitates the use of cadaver allograft as a temporary cover. Sequential autografting may increase a subject’s anxiety and fear due to the need for multiple surgical procedures with associated pain and prolonged hospitalization [27].

Autografting can provide wound closure, but can also result in serious consequences related to iatrogenic donor site wounds created during the surgical excision of the healthy autologous tissue. Donor site wounds can be extremely painful, result in a significant physiologic burden, dyspigmentation, and scarring and can convert to full-thickness wounds requiring management to provide definitive closure. Unfortunately, neither the final healed split-thickness autograft nor the healed donor sites are wholly normal skin in terms of thickness, elasticity, and strength, and even successful procedures result in a disfiguring scar. Thus, it is clinically meaningful to minimize or eliminate the need to harvest skin tissue for an autograft.

Both the healed, autografted wound sites and donor sites must undergo continuous, life-long physical and rehabilitative therapy to minimize scarring, release contractures, and promote long-term functionality of the healed wounds. Even with this intensive therapy, the resulting skin is often thin, sensitive, and easily damaged. Special precautions must be taken to maintain proper moisturization as well as effective sunscreen protection of the healed areas. Moreover, the grafted skin often develops contractures over time which in turn may result in wound reopening and/or limited mobility. Overall, the treatment regimen for complex skin defects is a cumbersome, painful process involving sequential surgical excision, temporary cadaver grafting, and autografting, and risks wholly unsatisfactory outcomes. Alternatives to the practice of donor site harvest and autografting for the treatment of severe burns and other complex skin defects are urgently needed for both adult and pediatric populations.

Surgeons studiously avoid autografting in pediatric patients due to the exposure to general anesthesia required for donor site harvest and its effect on development, and also the desire to minimize pain and the risk of donor site sequelae. The anesthetist’s role would include resuscitation, analgesia, sedation, anesthesia, and intensive care. Utmost care needs to be taken by providing adequate, early fluid resuscitation to maintain organ perfusion and control the extent of the burn injury itself. All burns require immediate cooling to halt the burning process; prolonged cooling of burns that are >15% total body surface area (TBSA) risks hypothermia in children. The burn should be covered with a sterile non-adherent dressing [17]. The surgeon must understand the physiologic derangements and subsequent anesthetic implications during severe burn injury treatment [28]. The most common complication is failure in wound closure resulting in local infection, underlying catabolism, or both. Infection can occur in the wound itself, donor sites, or in association with invasive vascular lines or catheters [17].

## 4. Clinical Need and Rationale

Every year in the United States, approximately 45,000 individuals experience burns that require them to be hospitalized, and of those individuals, approximately 10–20% require surgical intervention such as autografting [1,29,30]. Depending upon the extent of the burn, hospitalization can often be protracted. Based on data in the 2016 National Burn Repository Annual Report, which includes data collected from 96 hospitals from 36 states and the District of Columbia between 2006 and 2015, the average duration of hospitalization lengthens with increasing burn size by approximately 1 day or more for each percent TBSA burned [1]. In the pediatric population, scald and contact burns are the most common etiology in infants and younger children, whereas fire flame dominates amongst adolescents suffering burns [1]. Most pediatric burn injuries are approximately 20% TBSA, of which the majority of the wound is full-thickness [2]. Approximately 10,000 people in the United States die of burn-related infections every year [30]. Amongst those aged 1–17 in the U.S. in 2015, fire and burns were the sixth leading cause of hospitalizations and transfers for non-fatal unintentional injuries leading to hospitalization [4]. Fires and burns are currently the fifth leading cause of death in the United States that occur in the home, the third leading cause of unintentional injury-related fatality among children and adolescents aged 5–14, and the fourth most prevalent cause for infants and children aged 1–4 [5].

For the pediatric population, the harvest of autograft is undesirable. Infants and children possess thinner skin and represent unique challenges in both pain control and wound management during care of both the burn wound and donor sites. They typically have limited surface area from which to harvest autografts... In the United States, hospitalization of those who have sustained a ≤10% TBSA full-thickness or deep partial-thickness burn is aimed at monitoring and controlling donor site pain resulting from autograft harvest [31].

Given the seriousness of complex burns in both pediatric and adults populations, new therapies are needed that minimize or eliminate the need for autograft. Although advancements in the medical management of burns have been dramatic since the introduction of surgical wound debridement by Janzekovic in the 1970s [32], the SOC for burns remains the harvest of healthy skin from donor sites and its transplantation to the injury. At the 2014 International Congress on Pediatric Burns hosted by Shriners Hospital for Children and the Massachusetts General Hospital, the practice of donor site harvest was described as a barbaric procedure that urgently required new approaches and technologies. For over 30 years, burn care professionals have sought an alternative to autograft harvest and its concomitant transplantation. Skin substitute technologies to date have not provided the critical functions of intact human skin nor have they stimulated or restored the body’s endogenous repair capabilities. The critical function of a skin substitute is to achieve a thick, viable, epidermal layer that is firmly attached to a dermal matrix and thereby exerts “both mechanical and physiological effects by protecting the wound, maintaining microbial control, and hastening wound maturation” [33]. There is a need to generate an off-the-shelf living human skin substitute that is able to promote wound healing while limiting the harvest of healthy skin and reducing the creation of iatrogenic donor site wounds.

## 5. Summary of Currently Approved Products

Several products developed for the adult burn market have been shown to promote healing in pediatric burns, though many still necessitate autograft harvest and transplantation to achieve wound closure. Products used for the treatment of burn wounds in the pediatric population include animal collagen-derived dermal substitutes, cultured epithelial sheets, and bilayered skin substitutes.

Acellular dermal substitutes Biobrane^®^ and Integra^®^ have shown efficacy in the treatment of pediatric burn wounds. These products lack an epidermal layer but provide barrier function via a silicone membrane. Biobrane consists of a silastic silicone membrane bonded to a nylon membrane coated with peptides derived from porcine dermal collagen. Biobrane has been shown to be effective for the management of partial-thickness burns in children and superior to topical 1% silver sulfadiazine or beta-glucan collagen matrix in time to closure [34,35,36]. Integra is made from bovine collagen and shark cartilage glycosaminoglycan with a silicone membrane covering providing a barrier to water vapor loss. It is approved for use in life-threatening full-thickness or deep partial-thickness thermal burns in adults without sufficient autograft or with physiological conditions prohibiting autografting. Treatment of pediatric burns with Integra resulted in improved cosmesis in comparison to autograft-allograft treatment [37]. It has also been used successfully for other complex skin defects in pediatric subjects, including acute traumatic wounds and congenital abnormalities such as cutis aplasia [38]. Integra is a dermal substitute and does not possess an epidermal component, so patients must be autografted following treatment with Integra. Integra is slowly vascularized, which delays definitive wound closure with split-thickness skin graft (STSG). Furthermore, while the silicone membrane provides a barrier to water vapor loss, it has little effect on reducing the risk of infection. As with other products containing bovine collagen, however, there is increasing concern regarding the transmission of bovine spongiform encephalopathy (BSE). This skin substitute must be avoided in patients allergic to bovine products. The main disadvantage of this product is its lack of real and permanent epidermal components, which therefore results in difficulties to replace the dermal and epidermal layer simultaneously [39]. Unfortunately, there is a dearth of data analyzing the healing effects of Integra on burn wounds in a randomized, controlled setting [40]. Primatrix^®^ is a highly porous dermal matrix made from fetal bovine collagen, and is designed to provide wound coverage and promote inflammatory and mesenchymal cell recruitment, ultimately leading to revascularization of the wound bed [41,42]. The use of bovine collagen in this product leads to concern over the transmission of BSE. Primatrix lacks an epidermal component and does not provide barrier function. Furthermore, treatment with Primatrix must be combined with autologous skin grafting in order to achieve definitive wound closure.

AlloDerm^®^ and GraftJacket^®^ are made from decellularized human cadaveric skin tissues and the risk of disease transmission varies since each product lot is derived from a different human tissue donor. As a result of harvesting and limited testing, safety concerns including disease transmission and manufacture recalls due to suspect sterility results and donor screening procedures are associated with this type of allogeneic product [43,44,45]. Like Integra and Primatrix, neither AlloDerm nor GraftJacket possess an epidermal component and thus provide no barrier function. When used on full-thickness burns, AlloDerm was infiltrated with host cells, including endothelial cells, however, the take rate of STSG placed over AlloDerm was decreased compared to STSG alone [46].

Unlike acellular dermal substitutes, TransCyte^®^ is comprised of human fibroblasts grown on nylon mesh coated with porcine collagen and bonded to a silicone membrane. In its first pediatric burn trial, TransCyte treatment resulted in significantly shorter hospitalization than treatment with hydrodebridement and topical antimicrobials alone [47]. In the treatment of pediatric subjects with partial-thickness burns, TransCyte encouraged more rapid reepithelialization than treatment with Biobrane and reduced the need for subsequent autografting [48]. Despite encouraging results, TransCyte is not currently marketed.

Autologous keratinocyte cultures have also been used clinically in both adult and pediatric populations. Coverage of deep partial-thickness burns with cultured allogeneic keratinocytes has been shown to reduce scarring associated with these wounds [49]. Autologous keratinocyte cultures, such as Epicel^®^, consist of thin sheets of poorly-differentiated keratinocytes that contain no dermal component and provide no barrier function. A patient biopsy is subjected to a lengthy culture period of approximately four weeks to prepare Epicel, during which time the wounds must be managed temporarily by other methods. The resulting epidermal sheets are fragile and difficult to handle and have a shelf life of only 24 h. Epicel was approved by the Food and Drug Administration (FDA) in 2007 via Humanitarian Device Exemption for treatment of burns of >30% TBSA. It has been used successfully to treat large burn injuries in both adult and pediatric populations, though its effectiveness has not been shown in a clinical trial [50,51]. Since approval, 29% of individuals receiving Epicel were below 22 years of age [52]. The poor handling characteristics, intensive coordination for tissue harvest, expansion of the autologous keratinocytes, and short shelf life of Epicel compromise its utility in burn treatment.

Bioengineered skin substitutes composed of human keratinocytes growing on dermal analogs containing living human fibroblasts reproduce many structural and biological features of intact human skin. A recent study showed that engineered skin substitutes comprised of autologous keratinocytes and fibroblasts reduced mortality and donor skin harvest in the treatment of full-thickness burns of greater than 50% TBSA [53]. While these results are promising, the use of autologous cells requires their harvest from the patient, followed by a lengthy culture process to generate this custom treatment. This necessitates the use of temporary wound coverings such as cadaver allograft and a delay in definitive closure of the burn wound. Any delays in definitive closure increase the risk of infection and eventual scarring.

Two approved cellular products contain allogeneic keratinocytes and fibroblasts, alleviating the obligate temporal delay for the manufacture of autologous products. OrCel^®^ (Forticell Bioscience, New York, NY, USA) contains allogeneic keratinocytes and fibroblasts, however, the keratinocytes are not organized into a fully-stratified epidermal layer and therefore this product does not exhibit a competent epidermal barrier [54]. Further, this product is not currently marketed. The only currently-available, full-thickness, bioengineered, allogeneic skin substitute approved in the United States is Apligraf^®^ (Organogenesis, Canton, MA, USA). Apligraf contains keratinocytes harvested from human skin tissue that are grown atop a dermal analog composed of bovine collagen supplemented with living human fibroblasts [54,55]. It has a well-defined epidermal component that likely provides some barrier function, and use of up to three applications to the same site has shown success in the treatment of pediatric skin wounds due to epidermolysis bullosa, an inherited connective tissue disorder [56]. Apligraf is indicated as a second line treatment for chronic skin wounds after first-line therapies fail. Infection was a major adverse event in clinical trials on venous stasis ulcers; 29.2% of subjects receiving Apligraf had a suspected wound infection versus 14.0% in the control arm [57]. Although not approved in this indication, it has been used for the management of severe burns in adults [58,59]. Well-documented disadvantages of Apligraf include its poor handling characteristics and the fact that it rapidly disintegrates once placed in the wound bed, taking on an appearance which can be misinterpreted as wound infection [60]. Additionally, the manufacturing costs of Apligraf will necessarily remain high, as the human cells for Apligraf must regularly be sourced and require costly adventitious agent testing for each new cell bank. Furthermore, Apligraf has a short shelf life of 15 days [61].

## 6. Conclusions and Future Prospects

A definitive objective is to accomplish a biologic skin substitute containing both epidermal and dermal components that gives a successful outcome without the need for autografting and resultant pain and scarring. As a result of the limitations described above, there is a significant medical need for the clinical development of innovative, next generation, off-the-shelf therapeutic bilayered skin substitutes with a long shelf life that recapitulate the normal barrier function of the intact human skin and stimulate wound repair and skin regeneration. The identification of near-diploid immortalized human keratinocytes (NIKS) as a continuous, genetically uniform source of human keratinocytes is a promising discovery, and is currently undergoing clinical research. NIKS keratinocytes must be differentiated in a definitive way to produce a thick, suturable, skin substitute with both dermal and epidermal compartments that is amenable to mechanical meshing prior to patient placement. The biologic skin tissue substitute must not be patient-specific but rather a universal allogeneic skin substitute that reproduces many of the structural and biological properties of normal human skin. In addition, it must provide barrier function comparable to intact human skin with good handleability.

The biologic skin tissue substitute would be developed without any antigen-presenting cells such as Langerhans cells, lymphocytes, macrophages, dermal dendritic cells, or endothelial cells. Since the cells in the biologic skin tissue substitute would be replaced by the patient’s keratinocytes over time as they become capable of resurfacing the wound area, this replacement would be a direct result of an active wound healing environment, as the patient’s own cells would replenish the wound area, consistent with a native skin healing response, thus promoting autologous tissue regeneration.

In summary, the use of NIKS keratinocytes, an unlimited, consistent, and safe source of human cells, to generate this skin substitute tissue offers unprecedented manufacturing cost advantages versus other human skin substitutes which require ongoing pathogen and adventitious agent testing. Skin tissue of this kind presents significant advantages over currently available products used for treating complex skin defects, including the absence of bovine collagen, which reduces the risk of BSE transmission, and the production of bioactive molecules from this living tissue, including elevated levels of antimicrobial peptides such as human β-defensin-3 compared to skin substitutes prepared from other sources of human keratinocytes [62].

This kind of new technology might promise to significantly increase the therapeutic and commercial value of the cultured skin substitute as a first-line therapy in the treatment of burns by reducing surgical autografting in pediatric patients.
